# Endoscopic Assessment of Membrane Perforation During Osseodensification-Mediated Sinus Elevation in Sites With a Residual Bone Height (RBH) of 4-5 mm: A Pilot Study

**DOI:** 10.7759/cureus.117808

**Published:** 2026-10-12

**Authors:** Carel Ettel, Amine Choueiri, Gabriel Menassa, Zeina Majzoub

**Affiliations:** 1 Department of Periodontology, Saint Joseph University, Beirut, LBN

**Keywords:** dental implant(s), endoscopy, osseodensification, sinus membrane perforation, transcrestal sinus floor elevation

## Abstract

Background

The rate of sinus membrane perforation (SMP) during osseodensification-mediated transcrestal sinus floor elevation (OD-TSFE) has been assessed in a small number of clinical studies lacking diagnostic validity. Limited information is available regarding the rate of perforations under endoscopic monitoring. The objectives of this study are to assess: (i) the incidence, characteristics, and timing of occurrence of perforation during endoscopically monitored graftless OD-TSFE with simultaneous implant placement performed by a trainee periodontist; and (ii) inter-method reliability among three clinical perforation detection approaches.

Methods

A prospective cohort pilot study was conducted on patients requiring single implant-supported crowns in sites with residual bone height (RBH) of 4-5 mm. Sinuses with a flat sinus floor profile, narrow bucco-palatal width, and membrane thickness <2 mm were included. All OD-TSFE procedures with simultaneous implant placement were performed by a trainee periodontist according to the manufacturer’s protocol II. The incidence, timing, and characteristics of perforations were monitored intraoperatively using endoscopy, tactile evaluation, and direct visualization through the osteotomy using magnifying loupes. Six OD-TSFE procedures in five patients were included.

Results

All implants were 8 mm in length and had a mean of 3.4 ± 0.4 mm protruding into the sinus. No perforations were detected using endoscopy and direct visualization, while tactile evaluation suggested one perforation.

Conclusion

The small sample size and the actual absence of perforations precluded a meaningful comparative analysis between the three SMP detection approaches. Within the limitations of this pilot study, graftless OD-TSFE performed by a trainee periodontist under non-challenging anatomical conditions was not associated with any endoscopically detectable perforation.

## Introduction

In edentulous maxillary posterior sites, low bone density is frequently coupled with insufficient bone height for the placement of implants with the necessary length and primary stability. Sinus floor elevation (SFE) procedures are commonly used to treat volumetric deficiencies in the atrophied posterior maxilla. Among the various approaches to SFE, transcrestal sinus floor elevation (TSFE) has emerged as a minimally invasive procedure with reduced chair time, costs, discomfort, and rate of sinus membrane perforations (SMPs) when compared to conventional lateral SFE [1-4]. Furthermore, when comparing patient-reported outcomes following various SFE surgical approaches, the graftless transcrestal elevation seems to be the technique associated with the least post-operative morbidity and inflammation [5].

In 2013, the osseodensification (OD) technique was introduced by Huwais [6] as a novel biomechanical approach based on non-subtractive bone instrumentation in both osteotomy site preparation and TSFE. By compacting trabecular bone laterally and generating a hydrodynamic wave at the tip of the specially designed Densah® burs (Versah, LLC, Jackson, MI, USA) during drilling in counter-clockwise mode, OD facilitates controlled gradual detachment and elevation of the sinus membrane while enhancing primary implant stability [7,8].

SMP is the most commonly reported intra-surgical complication of SFE techniques [9]. Increased SMP prevalence has been reported to be associated with a higher risk of implant failure and reduced implant survival rates [10-12]. In general, SMP is directly related to the stretching potential of the Schneiderian membrane, which is affected by membrane health conditions, membrane thickness, sinus anatomic contours, bucco-palatal sinus width, presence of intra-sinus bony septa, subsinus residual bone height (RBH), membrane elevation height/implant protrusion length within the sinus, type of sinus elevation surgical procedure, use of bone grafting materials, physical properties of the grafts, and smoking [8,11-21]. When specifically considering TSFE, most of the above-mentioned confounding variables are likely to affect the clinical incidence of SMP [12,13,22,23]. In addition, the occurrence of SMP during TSFE is significantly affected by the type of instruments used to breach the osseous sinus floor and elevate the Schneiderian membrane [12,24].

A major limitation of the TSFE approach lies in its blind nature and the difficulty of accurately assessing intra-operative occurrence of SMP. Although OD has been reported to be associated with a low incidence of perforations [13], the available clinical evidence remains limited. Only four prospective clinical studies have reported on the occurrence of SMP using graftless OD-mediated TSFE [13,22,25,26]. Three out of these four studies relied on subjective SMP detection methods with inadequately documented diagnostic accuracy, such as direct visual inspection under magnification, injection of normal saline into the site, and immediate post-operative CBCT [13,22,25]. The fourth study by Gandhi [26] is the only investigation that evaluated SMP under endoscopy, which is considered the gold standard method for detecting SMP in TSFE. No detailed documentation was, however, provided by the author [26] relative to baseline sinus characteristics, which are key risk factors for perforations.

The aim of this prospective pilot study was to assess the occurrence of SMP during graftless OD-mediated TSFE with simultaneous single implant placement in posterior maxillary sites with 4-5 mm subsinus bone height, using direct endoscopic monitoring. Thus, the primary objective was to evaluate the occurrence of SMP during the procedure in sinuses with comparable dimensions, floor contours, and sinus membrane thickness. The secondary objectives were to assess implant primary stability, the size and timing of occurrence of potential perforations, and the inter-method reliability among three clinical SMP detection approaches (tactile evaluation, direct visualization using magnifying loupes, and endoscopic assessment). The inclusion of anatomically comparable sinuses allows exclusion of the impact of sinus-related risk factors on the incidence of SMPs, thereby restricting the cause-effect relationship to the surgical technique itself.

## Materials and methods

Study design and settings

The present study was designed as a single-center prospective cohort pilot study involving a group of patients with comparable sinus characteristics undergoing endoscopically monitored OD-mediated TSFE performed by a single trainee periodontal resident (C.E.) at the Department of Periodontology, Faculty of Dental Medicine, Saint-Joseph University of Beirut, Lebanon.

The study was approved by the Institutional Ethics Committee of Saint-Joseph University of Beirut (CER-2025-100). Registration was made at ClinicalTrials.gov under the ID number NCT07542275. 

Study population

The study population included systemically healthy patients requiring single implant-supported crowns in maxillary posterior sites who were recruited from the patient population attending the postgraduate Department of Periodontology, Faculty of Dental Medicine, Saint-Joseph University of Beirut. Patients and sites were considered eligible if they met the following inclusion criteria: age >18 years; unilateral or bilateral one-tooth edentulous site distal to the maxillary first premolar; the site could be either bounded on both proximal sides by natural teeth or bounded by an adjacent tooth on one side and an edentulous space on the other; healed edentulous sites with at least nine months following tooth extraction or loss; relatively flat and regular sinus floor contour; sinus bucco-palatal width (distance between the buccal and palatal walls at 10 mm apical to the residual alveolar crest) ranging from 8 to 12 mm [27]; sinus membrane thickness <2 mm; and subsinus RBH of 4-5 mm at the intended implant sites, with sufficient bucco-palatal ridge width to allow implant placement without the need for additional bone augmentation procedures.

The exclusion criteria included absolute contraindications for implant surgery [28], pregnancy or lactation, ongoing or previous conditions or medications affecting bone metabolism, ongoing or history of head and neck irradiation, oral inflammatory and autoimmune diseases, presence of osseous lesions or persistent infections at the planned implant sites, previous bone augmentation surgery at the intended implant sites, heavy smoking (>10 cigarettes/day), presence of intra-sinus bony septa in line with the planned implant sites, history of sinus surgery, and ongoing rhinitis or sinusitis.

A baseline pre-operative cone-beam computed tomography (CBCT) was used to verify patient- and site-related eligibility criteria and sinus conditions. 

Sample size calculation

The sample size was not calculated based on the primary outcome variable, occurrence of SMP. Instead of adopting a power-based sample size, the authors applied a convenience sampling approach and included all eligible patients who could be enrolled during a set time frame of seven months (September 1, 2025-March 24, 2026) due to the fixed study duration and the stringent patient inclusion criteria. Consequently, the present study, based on a feasibility-based approach, is considered a pilot study.

Interventions

All patients underwent a thorough pre-operative evaluation and a comprehensive treatment plan as needed prior to implant placement. Full-mouth scaling and oral hygiene instructions were provided prior to surgery. In the presence of untreated periodontitis, patients received full-mouth scaling and root planing, followed by re-evaluation at three months to ensure the establishment of periodontal health.

Prior to anesthesia (2% articaine with 1:100,000 epinephrine), patients were asked to rinse with a 0.12% chlorhexidine solution for one minute, followed by facial skin antisepsis with chlorhexidine. A 5-mm antrostomy was positioned as apically as possible and in the closest proximity to the anterior wall of the maxillary sinus. The main flap was extended mesially with a vertical release incision, or a minimally invasive separate semi-lunar flap was used according to the mesio-distal position of the antrostomy relative to the planned implant site.

Following flap elevation, the antrostomy was created using a round diamond bur mounted on a straight high-speed handpiece under continuous sterile saline irrigation. After completion of the bony window, gentle aspiration was performed to exclude the presence of any low-density fluid undetected on baseline CBCT and, importantly, to decompress the sinus before proceeding. The Schneiderian membrane was then carefully incised using a 15C blade (Swann-Morton® Limited, Sheffield, England) to allow the insertion of a portable straight rigid endoscope (V102 HD 3-in-1 USB Endoscope Camera; Inskam; Shenzhen YiPinCheng Technology Co., Ltd., Shenzhen, China) into the sinus lumen. Once adequate visualization of the sinus floor at the intended implant site was achieved, the osteotomy was prepared using Densah® burs in accordance with the manufacturer’s recommendations for crestal sinus lift protocol II [8]. Briefly, the pilot drill was used to within 1 mm of the sinus floor. The 2.0-mm bur was then used in counterclockwise mode at 1000 rpm with copious irrigation, up to the level of the dense sinus floor. Subsequently, the 3.0-mm bur was advanced to the sinus floor in counterclockwise mode using a gentle pumping motion. Upon sensing haptic feedback indicating contact with the sinus floor, the bur was advanced beyond the floor in delicate 1-mm increments. The final osteotomy was completed using either the 3.3-mm or 4.0-mm bur in densifying mode, according to the bone quality at the recipient site, and advanced to a maximum depth of 3 mm beyond the sinus floor to elevate the Schneiderian membrane. Straumann® Bone Level Tapered implants (BLT™, Institut Straumann AG, Basel, Switzerland), a length of 8 mm, and a diameter of 4.1 mm according to ridge width were placed, and healing abutments were connected.

Continuous endoscopic monitoring was maintained throughout the entire procedure (implant site preparation and implant placement), with simultaneous video recording and photographic documentation, enabling real-time assessment of the Schneiderian membrane status and detection of any SMP by the clinician handling the endoscope (A.C.). To minimize observational bias, the operating surgeon (C.E.) was blinded to the endoscopy LCD monitor and independently assessed sinus membrane status at the end of each drilling step using tactile evaluation with a sinus probe and direct visualization with magnifying loupes equipped with a light source at power factors of 3× and 6× (Hyperloupe TTL, Hyperloupe™, Beirut, Lebanon). After completion of sinus membrane status assessment with these two evaluation methods, the findings of endoscopy were disclosed to the operating surgeon. When endoscopically detected, an SMP was managed based on its size and timing of occurrence, according to the protocol of Tavelli et al. 2020 [29].

The antrostomy site was covered with a resorbable collagen membrane (CollaTape®, Zimmer Biomet), and the flaps were tension-free sutured using 5-0 coated PGA resorbable sutures (DemeTech Co., Miami Lakes, Florida, USA) to achieve primary closure.

All surgical procedures were performed by the resident (C.E.) under the supervision of an experienced periodontist proficient in OD-mediated TSFE (A.C.).

Preemptive analgesia was achieved by administering ibuprofen 600 mg or paracetamol 1000 mg in patients with contraindications to nonsteroidal anti-inflammatory drugs (NSAIDs) 30-60 minutes prior to surgery. Antibiotic prophylaxis consisted of amoxicillin 1 g or clindamycin 300 mg (in case of penicillin allergy) every 12 hours, initiated 24 hours pre-operatively and continued for six days post-operatively. Post-operatively, paracetamol 1000 mg was recommended every eight hours as needed for pain control. Patients were instructed to rinse with a 0.12% chlorhexidine mouthwash twice daily for one week, starting the day after surgery. Sutures were removed 14 days post-operatively.

Investigated variables

Outcome Measures

The primary outcome measure was the endoscopically recorded intra-operative incidence of SMP. The secondary outcome measures included timing of membrane perforation using tactile evaluation and direct visualization (specific drilling step during which the SMP occurred) and endoscopic monitoring (drilling step or implant placement); type and size of membrane perforation classified as pinhole perforation, small perforation (≥1 mm and <2 mm), or large perforation (<2 mm) using endoscopic monitoring and direct visualization; and insertion torque value (ITV) measured at implant placement using a calibrated manual torque wrench. Maximum ITV was recorded during the final ¼ turn prior to reaching the planned final insertion depth and was rounded to the closest 5 Ncm.

Independent Variables

The following independent variables were recorded: patient’s age; sex; smoking status; RBH (recorded on pre-operative bucco-palatal CBCT image as the shortest distance from the alveolar crest to the lowest point of the maxillary sinus floor); bone quality assessed subjectively according to the classification of Lekholm & Zarb (1985) (Type I, Type II, Type III, and Type IV) [30]; and implant protrusion length (measured as implant length minus RBH). 

Statistical analysis

Data were organized and analyzed using spreadsheet-based statistical processing. Categorical variables, including patient’s sex, implant sites, tactile bone quality, occurrence of SMP, timing, and type and size of SMP, were expressed as frequency and percentage distributions. Continuous variables, including patient’s age, RBH, and ITV, were summarized using mean, standard deviation (SD), and range values.

The three SMP detection techniques (endoscopic monitoring, tactile evaluation, and direct visualization) were compared with respect to SMP occurrence (dichotomous binary outcome (yes/no)) using the non-parametric Cochran’s Q test, which is used to compare three or more paired proportions when the sample size is small.

## Results

A total of six OD-mediated TSFE procedures in five patients were included in the present analysis. All surgical sites healed uneventfully, with no sinus adverse events recorded up to two months following surgery (Figure 1).

**Figure 1:**
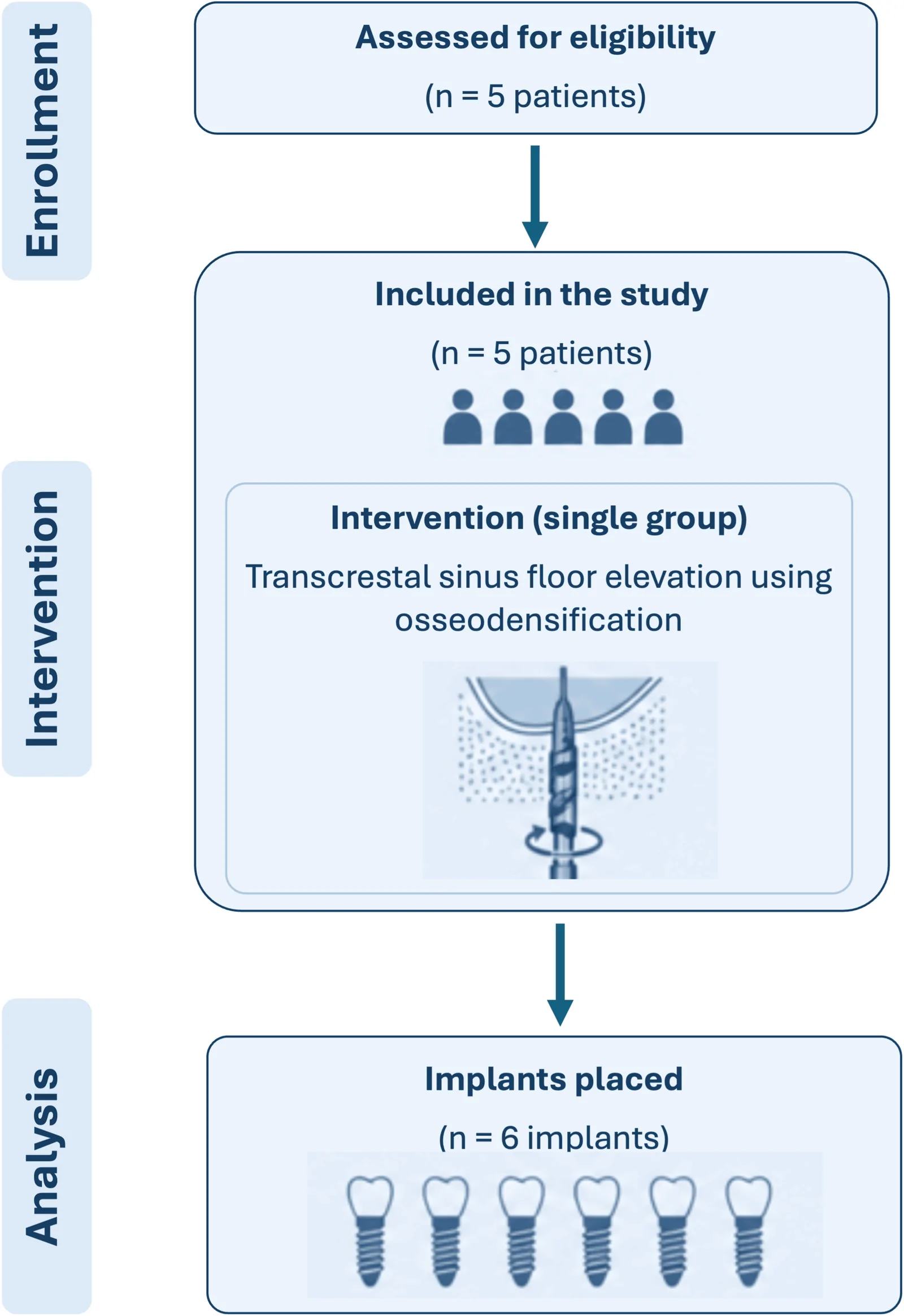
Flow diagram of patient enrollment, intervention, and implant placement in the single-group pilot study This image is an original author-created schematic using Microsoft PowerPoint (Microsoft® Corp., Redmond, WA, USA).

Patient- and site-related data are summarized in Table 1. 

**Table 1:** Characteristics of patients, sites, and implants included in the study M: male; F: female; NS: non-smoker; S: smoker; RBH: residual bone height; ITV: insertion torque value

	Age	Sex (M/F)	Smoking status (NS/S)	Implant location	RBH (mm)	Implant protrusion length (mm)	Bone quality	ITV (Ncm)
Patient 1	47	F	S	16	4	4	III	40
Patient 2 Implant 1	53	M	S	15	4.3	3.7	III	40
Patient 2 Implant 2	53	M	S	17	4.6	3.4	III	35
Patient 3	36	M	S	26	4.7	3.3	III	40
Patient 4	61	M	NS	27	5	3	IV	25
Patient 5	51	M	S	16	4.9	3.1	III	40
	49.6 ± 9.2	M = 80%, F = 20%	S = 80%, NS = 20%	-	4.6 ± 0.4	3.4 ± 0.4	III = 83.3%, IV = 16.7%	36.7 ± 5.9

No membrane perforation was endoscopically detected in any of the six procedures at any intra-operative stage. Tactile feedback suggested one SMP, whereas magnification-based assessment revealed no SMP in any of the cases.

Endoscopic images showed membrane elevation resembling a flattened dome with circumferential membrane detachment (Figure 2). During video recordings of the drilling procedures, the progressive stretching of the membrane was associated with a change from a normal pink appearance with visible superficial vessels to a localized whitened elevated area with some superficial reduction in vascular visibility. Following implant placement, a localized bluish-gray translucency through the intact membrane was evident at the center of the elevated site in two cases, while the whitish pale appearance with less distinct superficial vascular network remained unchanged at four sites.

**Figure 2:**
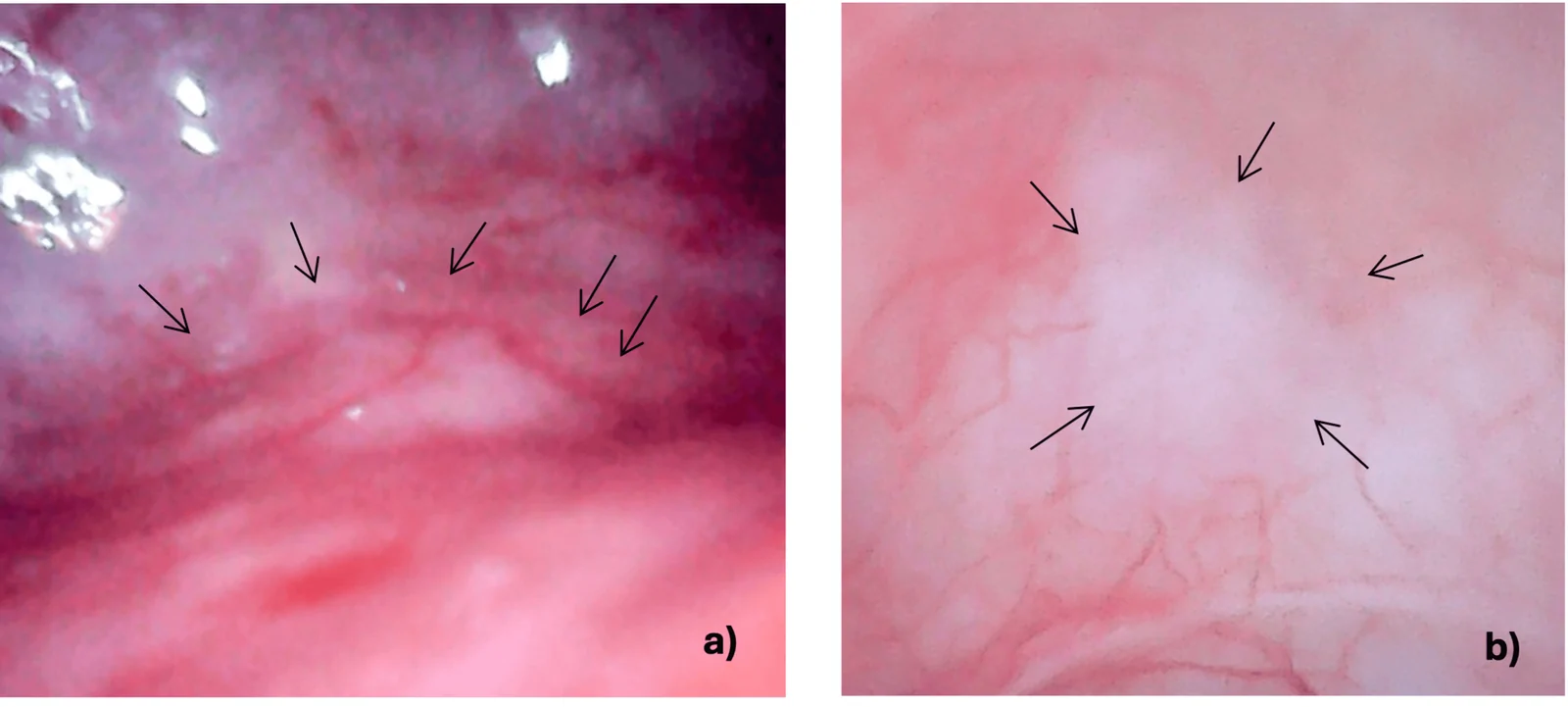
Endoscopic views of the sinus membrane with no evidence of perforation following implant placement a) Flat-dome-like elevation of the membrane (black arrows); b) Elevated membrane with whitish appearance and less visible superficial vascular network.

SMP occurrence could not be statistically compared across the three detection modalities (endoscopic, tactile, and magnification-based assessment) and is reported descriptively instead. Although Cochran’s Q test represents the appropriate non-parametric method for comparing related proportions, inferential analysis was not carried out due to the small sample size and the sparse distribution of SMP events. Under these conditions, the assumptions underlying asymptotic statistical testing were not satisfied, rendering p-values unreliable and potentially misleading. 

## Discussion

The aims of the present prospective pilot cohort study were to investigate, under endoscopic monitoring, the rate, timing, and characteristics of SMP occurring during graftless OD-mediated TSFE with simultaneous implant placement. The current literature on the incidence of SMP during TSFE using densifying burs remains inconclusive. This is largely due to the scarce number of clinical studies that used detection methods with high diagnostic accuracy and real-time assessment of membrane integrity, such as intra-operative endoscopy. To the authors’ knowledge, the present clinical study is only the second to investigate SMP occurrence in graftless TSFE using the OD approach under endoscopy since the initial report by Gandhi [26].

All six procedures in the present study were completed with zero incidence of SMP. These findings are consistent with the low rate of perforations (1 in 10 cases) reported by Gandhi [26]. However, it should be noted that the cohort sample in the present study had maxillary sinuses with standardized baseline anatomical characteristics, i.e., flat sinus floor profile, narrow bucco-palatal sinus width, membrane thickness <2 mm, and a limited range of RBH between 4 and 5 mm, in contrast with the absence of clearly defined sinus-related inclusion criteria and the anatomical heterogeneity within the patient sample treated in Gandhi’s study [26]. In addition, only single implants were placed with implant protrusion lengths in the sinus limited to a small range of values (3-4 mm). These stringent inclusion criteria adopted in the present investigation likely reduced the influence of anatomical confounders, strengthened the internal validity of the study, and restricted the cause-effect relationship primarily to the surgical technique itself.

Most studies assessing SMP in OD-mediated TSFE relied on indirect detection methods, such as post-operative periapical radiographs [31], post-operative CBCT [25,32], and the saline injection test [25]. No SMPs were reported in the three abovementioned studies, in line with the results of the present study. Similarly, Huwais et al. found no SMPs in a multicenter retrospective study analyzing 261 implants placed in 222 patients with RBH ≥2 mm using graftless and grafted OD-mediated TSFE [33]. It should be emphasized that the absence of a clearly defined detection method in Huwais et al.’s study limits the interpretation of these findings. In contrast, the study of Mazor et al., which evaluated grafted and graftless TSFE with OD protocols in RBH ≥2 mm and ≤6 mm, showed an SMP incidence of 7.3% using magnification for direct visualization of sinus membrane integrity [13]. This higher rate of SMP may be attributed to the inclusion of 165 sites with RBH ≤3 mm, excessive vertical membrane elevation beyond 4 mm, and the use of long implants (10-12 mm) in sites with limited RBH (2-3 mm), all factors associated with a higher perforation risk [34,35].

The low or zero rate of SMP during TSFE with the densifying burs can be explained by the biomechanical and hydrodynamic characteristics inherent to OD, whereby the pressure wave generated apical to the bur tip, coupled with the use of the bur in a bouncing motion, allows controlled transcrestal breach of the sinus floor and detachment of the sinus membrane [7,8,36,37]. This effect is comparable to a “membrane pre-detachment prior to elevation” previously shown to significantly reduce the risk of perforation during TSFE [38]. Another factor that can explain the lack of SMP in the present study is the endoscopically verified horizontal detachment of the sinus membrane circumferentially to the implant perimeter associated with a relatively small vertical elevation. This pattern may reduce the risk of SMP, as reported by Sonoda et al. [39]. In addition, the graftless approach used in the present study may have also contributed to the zero incidence of SMP, as the physical properties of graft particles and their distribution may create uneven pressure points and significantly affect the risk of membrane tearing, especially in thin, stretched membranes [19].

The endoscopic observations of color changes of the Schneiderian membrane during the drilling sequence are likely to reflect the biomechanical and microvascular responses of the membrane during elevation. As the membrane is elevated, it becomes progressively thinner, altering light transmission, tissue translucency, and visibility of the underlying structures. In addition, the superficial vascular plexus of the membrane may become compressed, stretched, or displaced laterally during elevation, leading to a whitened appearance, often coupled with the disappearance of fine superficial vessels. This phenomenon is similar to soft tissue blanching elsewhere under pressure. Following implant placement, the thinned membrane becomes semi-transparent, allowing visualization of the underlying structures. When considering the relationship between the implant apex, the elevated sinus membrane, and the autogenous bone particles generated during the counterclockwise drilling sequence, bone particles can either spread laterally within the elevated subantral compartment or remain densely concentrated around the implant and above the implant tip (Figure 3). The bluish-gray translucency through the intact membrane observed following implant placement is probably related to the implant apex supporting the elevated membrane directly, with little or no intervening buffer layer of autogenous bone particles.

**Figure 3:**
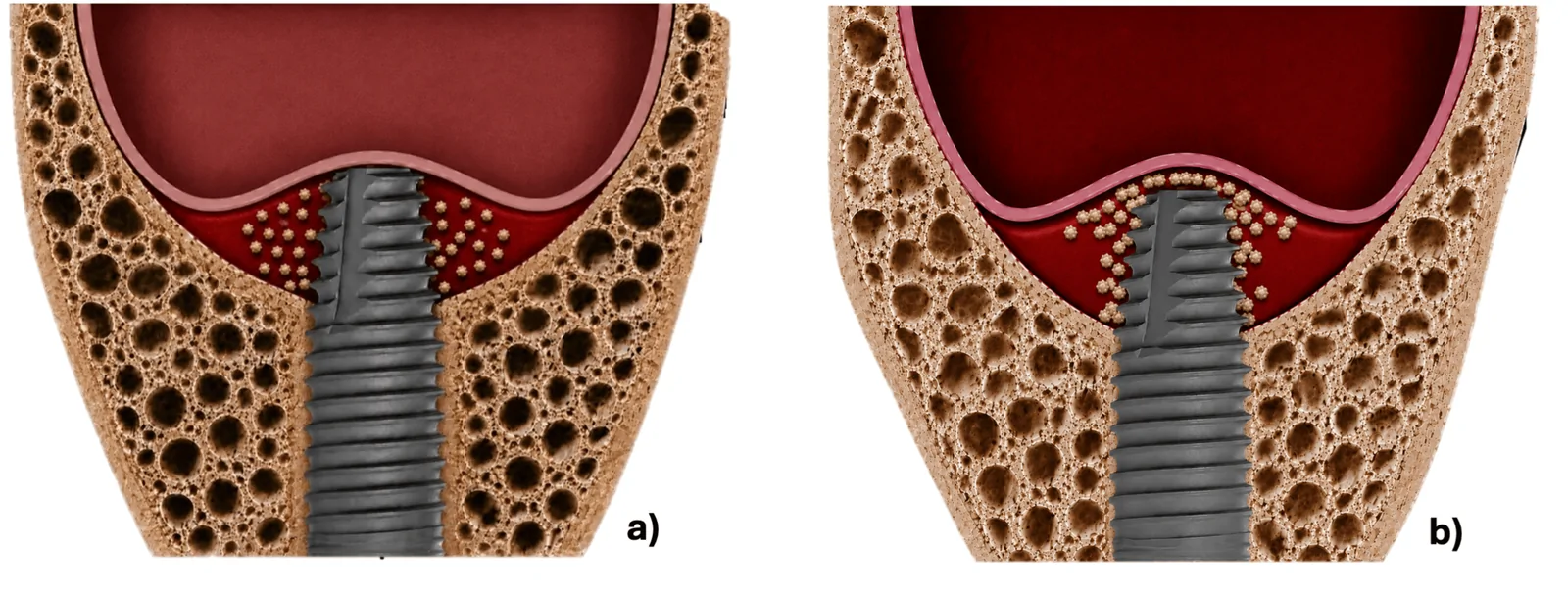
Schematic illustrations showing two possible scenarios of the relationship between the implant apex, the elevated sinus membrane, and the autogenous bone particles generated during the counterclockwise drilling sequence a) Bone particles spread laterally within the elevated subantral compartment and the elevated sinus membrane is directly supported by the implant apex; b) bone particles are densely concentrated around the implant and above the implant tip. The compacted apical mass of autogenous bone chips provides support to the elevated membrane.

The secondary objectives of the present study aimed to compare magnification-based and tactile approaches versus endoscopy with respect to their diagnostic accuracy in detecting SMP. Although magnification-based assessment and endoscopic visualization yielded concordant findings with no perforations identified, this agreement cannot be interpreted as evidence of diagnostic validity. In contrast, tactile evaluation detected one perforation that was not confirmed endoscopically, indicating occasional overestimation due to the subjective nature of tactile feedback.

OD has been consistently shown in clinical studies and systematic reviews to achieve improved primary stability when compared to subtractive drilling in osteotomy site preparation [40-43] and osteotomes in TSFE procedures [3,44-46]. In the present study, implant placement in RBH of 4-5 mm with relatively poor bone quality (Types III and IV) achieved a mean insertion torque of 36.7 ± 5.9 Ncm, in agreement with the ITV value of 32.9 ± 6.7 Ncm recently reported by Kafadar-Gürbüz et al. in RBH of 4 to 6 mm and a combination of Types III and IV bone quality [44]. 

In the present study, the clinician who performed the TSFE procedures was a trainee periodontist with limited experience in OD. Despite the favorable sinus anatomical and physiological conditions in the treated sites, TSFE remains a technique-sensitive, blind procedure heavily dependent on tactile sensitivity. Less experienced clinicians are likely to have reduced ability to detect subtle tactile changes and less instrument control, increasing the risk of over-drilling and membrane perforation. The absence of SMP in the present study is likely attributable not only to the non-challenging anatomy of the treated sites but also to the “user-friendly” controlled nature of OD and its relatively easy learning curve. Of particular relevance, the trainee surgeon was able to achieve high primary stability equivalent to that reported in investigations conducted by experienced surgeons [44]. This highlights the fact that trainee periodontists can achieve clinical outcomes comparable to experienced surgeons when operating under supervision and following well-defined protocols.

While the present pilot study was designed to maximize internal validity, certain limitations must be considered when interpreting its findings. The limited sample size restricts statistical power, particularly for detecting low-frequency events. Furthermore, the rigorous inclusion criteria applied for the selection of sinuses with comparable anatomy, although essential to minimize the impact of anatomical confounding variables, limit the generalizability of the results to more complex anatomical conditions that are more frequently encountered in routine clinical practice. Accordingly, the absence of SMP in the present study does not imply a null risk in all clinical scenarios of OD-mediated TSFE. The present findings should also be interpreted within the specific context of graftless OD-mediated TSFE and may not be directly extrapolated to graft-assisted procedures. Finally, from a study design perspective, the absence of a comparative control group, such as conventional osteotome-mediated or other TSFE approaches, limits the ability to establish causal superiority between techniques and identify the technique with the lowest SMP rate. 

In the present study, the use of a straight rigid endoscope to monitor sinus membrane integrity was dictated by availability considerations. Such traditional endoscopes present significant challenges due to their limited field of view and restricted maneuverability, which necessitate enlargement of the antrostomy to ensure adequate access and visualization of the sinus floor. As an alternative, articulating rigid-flexible or angled rigid endoscopes are likely to improve intra-luminal navigation and visibility, allow for a smaller and less invasive access, and facilitate superior photographic documentation by allowing different shooting angles and improved three-dimensional perspective.

## Conclusions

Endoscopic monitoring of OD-mediated graftless TSFE with simultaneous implant placement, performed by a trainee periodontist in non-challenging sinus anatomy and with RBH of 4 to 5 mm, demonstrated a 0 incidence of SMP, supporting the potential of OD as a safe and predictable procedure for TSFE under controlled conditions. As a pilot study, the present investigation was primarily intended to provide preliminary data under specific clinical conditions rather than definitive conclusions of a negligible perforation risk applicable in various anatomic scenarios. This limits the study’s external validity and underscores the need for larger, controlled clinical studies incorporating diverse anatomical conditions to validate and extend these findings.
